# Quercetin Preventive Effects on Theophylline-Induced Anomalies in Rat Embryo

**DOI:** 10.17795/jjnpp-17834

**Published:** 2014-08-01

**Authors:** Neda Sistani Karampour, Ardeshir Arzi, Hossein Najafzadeh Varzi, Babak Mohammadian, Mohsen Rezaei

**Affiliations:** 1 Department of Pharmacology and Toxicology, School of Pharmacy, Jundishapur University of Medical Sciences, Ahvaz, IR Iran; 2 Department of Pharmacology and Toxicology, Physiology Research Center, School of Pharmacy, Jundishapur University of Medical Sciences, Ahvaz, IR Iran; 3 Department of Basic Sciences, Faculty of Veterinary Medicine, Shahid Chamram University, Ahvaz, IR Iran; 4 Department of Pathobiology, Faculty of Veterinary Medicine, Shahid Chamram University, Ahvaz, IR Iran

**Keywords:** Theophylline, Quercetin, Malondialdehyde, Glutathione peroxidase

## Abstract

**Background:**

Theophylline has been shown to cause heart anomaly in animal and human fetus.

**Objectives:**

The present study aimed to investigate the protective effect of quercetin on theophylline-induced heart disorders in rat embryo.

**Materials and Methods:**

Theophylline-induced teratogenicity in rats was used as the animal model. Pregnant rats were administered theophylline (259 mg/kg, po) or theophylline plus quercetin (259 mg/kg, po and 100 mg/kg, ip, respectively) on 9th and 10th days of pregnancy. On day 19, cardiac changes were assessed, measuring malondialdehyde (MDA) and glutathione peroxidase (GPx) activity levels in blood samples and also the fetus heart weight. Histopathological examination was also performed on all specimens.

**Results:**

Theophylline-treated rats showed MDA level elevation and GPx activity reduction. Quercetin treatment improved heart conditions and resulted in a significant reduction in MDA levels and elevation in GPx activity. Moreover, co-administration of quercetin and theophylline increased the heart weight significantly. Furthermore, histophatological study showed no changes in the treated groups.

**Conclusions:**

This study demonstrated that quercetin have beneficial effects on theophylline-induced-anomalies in rat embryo.

## 1. Background

Congenital anomalies are among the most important causes of infant mortality. Genetic disorders and environmental factors, like maternal illness, receiving harmful chemicals and physical injuries during pregnancy, are of the most important causes of congenital anomalies. Certain medications are of the most prominent anomaly factors, during pregnancy (1). There are reports of anomalies in fetuses of mothers who had used theophylline during pregnancy, the most important of which are cardiovascular disorders (1, 2). Theophylline is a phosphodiesterase enzyme inhibitor, widely used for asthma (3).

The importance of theophylline in the treatment of asthma has decreased, due to more powerful effects of beta-receptor agonists and inhalation forms of anti-inflammatory compounds, in acute and chronic asthma, respectively. However, theophylline may be chosen as the third line treatment, in the absence of a good response to beta-receptor agonists or corticosteroids. It also of use in the treatment of patients with poor economic status and in countries with limited healthcare measures, due to its very low cost (3).

The most common complications of theophylline observed in asthmatic patients are restlessness, dizziness, sinus tachycardia, nausea, emesis and convulsion. It also easily crosses the placenta and harms the fetus (4, 5). Theophylline has dose-dependent teratogenic effects on the cardiovascular system development. Anomalies observed in infants born from mothers with asthma, using oral theophylline include double outlet right ventricle (DORV), heart murmur, aortic displacement, ventricular septal defect (VSD), pulmonary valve stenosis and hypoplasia of the left ventricle and the mitral valve (1, 2).

Theophylline-induced complications in laboratory animals (rabbits, rats and mice) include cleft palate, abnormalities in fingers, small organs, micrognathia, bandy legs, DORV, aortic displacement and VSD (6, 7).

Fetal anomalies may be attributed to oxidative stresses, increasing the level of free radicals. Weakness of antioxidant defense and reduced antioxidant enzyme activity, like superoxide dismutase and glutathione peroxidase, are some of the causing factors (8). Nowadays, antioxidant activity and the amount of body oxidized biological products can be determined, in order to evaluate oxidative stress. The amount of serum malondialdehyde is a general index of lipid peroxidation, growing with oxidative stress increase. Glutathione peroxidase level is used as an index for the evaluation of antioxidant activity (9).

Since some of the drug teratogenic effects are induced by free radicals and oxidative stress, a preventing solution to these conditions is to use antioxidants compounds. Quercetin, commonly named sophretin and meletin, is a herbal flavonoid found in abundance in apple, onion, tea, green tea leaf, strawberries, broccoli and other plants (10, 11). Quercetin applies its anti-inflammatory properties by inhibiting inflammatory mediators and enzymes, like lipoxygenase. Researches have indicated that quercetin slows down the cancer cell growth, making it effective in cancer treatment, especially prostate cancer (10). This substance inhibits histamine release from basophils and mast cells, therefore, it has anti-inflammatory properties and helps asthma and allergy control (10, 11). However, its most important property is the antioxidant effect. This substance reduces oxidative stress and destroys free radicals.

## 2. Objectives

Since quercetin has been proved to have significant antioxidant properties in many studies, the present study investigated its effects on macroscopic and microscopic cardiac anomalies, in rat fetus, to give a more precise answer to this question: how much the cardiac theophylline-induced anomalies are related to oxidative stress and how useful the antioxidants are in the reduction of these anomalies.

## 3. Materials and Methods

### 3.1. Theophylline Preparation

Theophylline was purchased from Daroopakhsh Company (Iran), dissolved in normal saline and prepared immediately, preceding its use.

### 3.2. Quercetin Preparation

Quercetin powder was purchased from Sigma Chemical Company (St. Louis, MO, USA)dissolved in 0.9 % normal saline, mixed vigorously and stored in a dark bottle at temperature of 4ºC. The quercetin solution was freshly prepared each day. 

### 3.3. Animals

The Wistar albino rats were obtained from the animal house of Jundishapour University of Medical Sciences, Ahvaz, Iran, and kept under specific conditions, on a constant 12-hour light/dark cycle, at a controlled temperature of 23 ± 2°C. Standard pellet food and tap water were available ad labitum. Mature female rats were mated overnight at a 3:1 female: male ratio. The pregnant female Wistar rats were then randomly divided into four equal groups (n = 10); a control group and three study groups. The test groups received theophylline (259 mg/kg, po), quercetin (100 mg/kg, ip) and theophylline plus quercetin (259 mg/kg, po and 100 mg/kg, ip), respectively on the 9th and 10th days of gestation. The control group received normal saline (0.5 mL/100g, po) on the 9th and 10th days of gestation.

On day 19, pregnant females from all groups (control and study) were anesthetized with ether and blood samples were collected from their hearts for biochemical assay. The offsprings were then recovered, number of absorbed fetuses was measured and their hearts were removed. All blood samples were centrifuged at 2500 rpm, for 15 minutes, at temperature of 4ºC. Serum samples were collected to assay the malondialdehyde and glutathione peroxidase levels.

### 3.4. Malondialdehyde Assay

Serum malondialdehyde (MDA) was determined according to the method of Buege and Aust, using bioassay laboratory ELISA kit (China). Standard and samples are aspirated into wells and the target protein is bound to specific immobilized monoclonal capture antibodies. The biotinylated secondary specific antibody is used for detection. To avidin-biotin-peroxidase complex, TMB substrate solution is added. The solution color changes after adding acidic TMB stop solution. Each step needs sufficient amount of washing.

### 3.5. Glutathione Peroxidase Assay

Serum glutathione peroxidase activity was assessed, according to the method of Paglia and Valentine, using Bioassay laboratory ELISA kit (China). Standard and samples are aspirated into wells, the target protein is bound to specific immobilized monoclonal capture antibodies. The biotinylated secondary specific antibody is used for detection. To avidin-biotin-peroxidase complex, TMB substrate solution is added. The solution color changes after adding acidic TMB stop solution. Each step needs sufficient amount of washing.

### 3.6. Histologic Techniques

The hearts were fixed in 10% buffered formalin, embedded in paraffin, sectioned stained with hematoxylin andeosin and E striated for microscopic studies.

### 3.7. Statistical Analysis

The data are expressed as mean ± SE. Statistical differences between means were determined by one-way analysis of variance (ANOVA), followed by LSD test and a threshold of significance of P < 0.05.

## 4. Results

**Figure 1.**
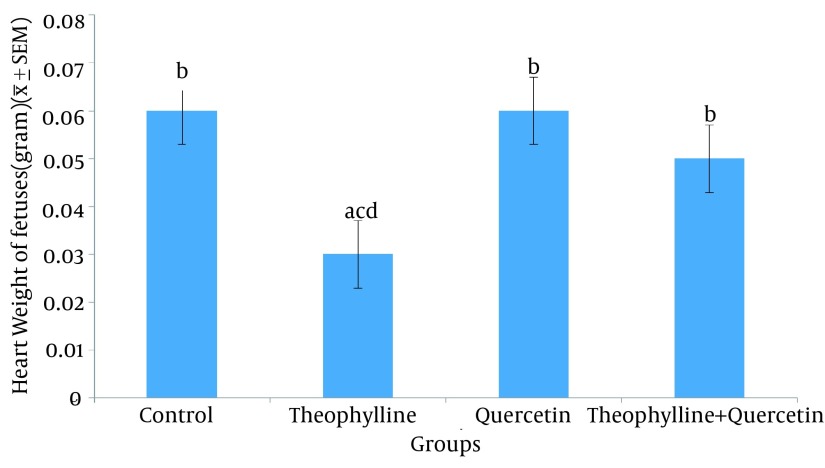
Heart Weight (Mean ± SE) of Fetuses in Control and Test Groups 1) Normal saline (control) (0.5 mL/100 g, po); 2) Theophylline (259mg/kg po); 3) Quercetin (100 mg/kg, ip); 4) Theophylline + quercetin (259 mg/kg, po;100 mg/kg, ip). Different letters show significant difference between groups (P < 0.05, n = 30).

No significant difference was observed in heart weights and the percentage of absorbed fetuses, between the groups receiving quercetin alone, quercetin plus theophylline and the normal saline group (Table 1 and Figure 1) 

**Table 1.** Number and Percentage of Live and Absorbed Embryos in Control and Test Groups

Groups	Number of Embryos	Percentages of Live Embryos, %	Percentage of Absorbed Embryos, %
**Normal saline**	68	98.53	1.47
**Theophylline**	37	58.20	41.8
**Quercetin**	53	94.33	5.66
**Theophylline + Quercetin**	61	85.25	14.75

**Figure 2.**
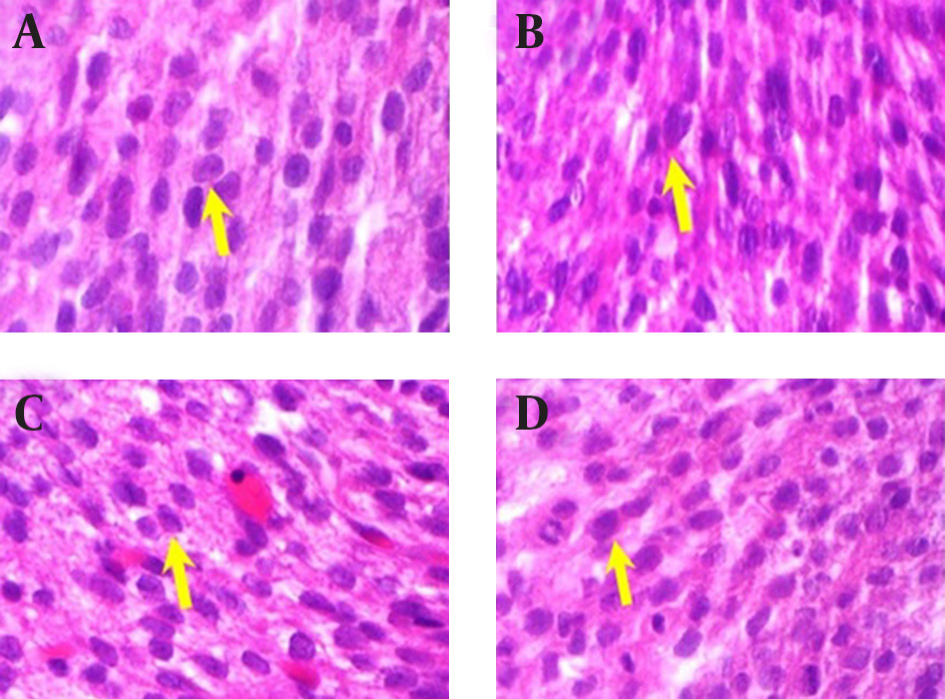
The Quercetin and Theophylline-Induced Histopathological Changes in Rat Heart A) Normal heart histology in the Saline Group; B) Heart histology in theophylline group; C) Heart histology in quercetin group; D) Heart histology in “theophylline + quercetin” group.

No apparent morphological or specific pathological changes were observed during the heart macroscopic and microscopic examinations of the rat fetuses, in the normal saline (control) group, the theophylline receiving group, the quercetin receiving group and the group receiving both quercetin and theophylline. Pericardium, bilobate and mitral valves and tricuspid and pulmonary valves were found to be normal. Cardiac muscle cells were also normal; their large purple nucleus was located in the center of their pink colored cytoplasm (Figure 2).

**Figure 3.**
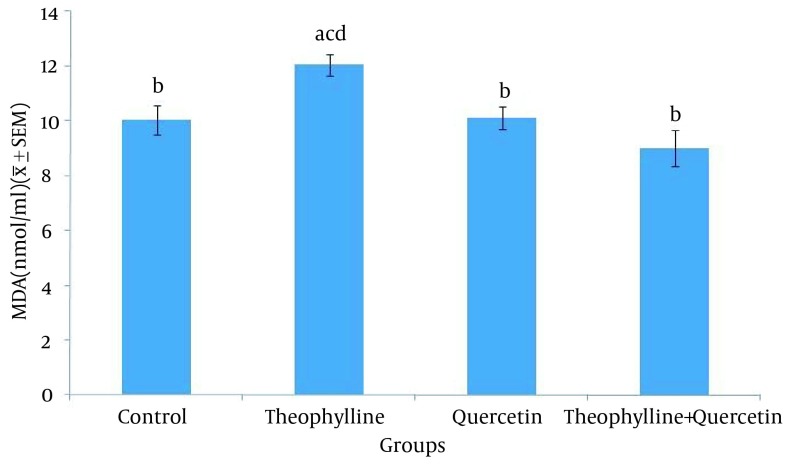
Serum Malondyaldehyde Level in Control and Test Groups 1) Normal saline (control) (0.5 mL/100 g, po); 2) Theophylline (259 mg/kg, po); 3) Quercetine (100 mg/kg, ip); 4) Theophylline + quercetin (259 mg/kg, po; 100 mg/kg, ip). Data are presented as Mean ± SEM. Different letters show significant differences between groups (P < 0.05, n = 30).

**Figure 4.**
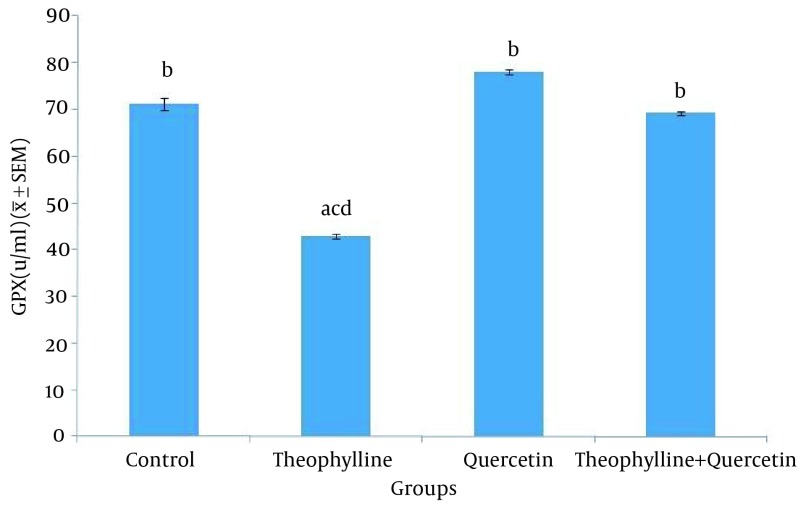
Serum Glutathione Peroxidase Level in Control and Test Groups 1) Normal saline (control) (0.5 mL/100 g, po); 2) Theophylline (259 mg/kg, po); 3) Quercetine (100 mg/kg, ip); 4) Theophylline + quercetin (259 mg/kg, po; 100 mg/kg, ip). Data are presented as Mean ± SEM. Different letters show significant differences between groups (P < 0.05, n = 30).

The average malondialdehyde level in rat serum was significantly higher (P < 0.05) in the group receiving theophylline, compared to other groups (Figure 3). The average glutathione peroxidase level in rat serum was significantly lesser (P < 0.05) in the group receiving theophylline, compared other groups (Figure 4).

## 5. Discussion

Promotion of free radicals and oxidative stress by medications is one of the main mechanisms of developing anomalies. The present research investigated the effect of quercetin (as an antioxidant factor) on the prevention of theophylline-induced heart anomalies in rat embryos. No significant difference was observed in the percentage of absorbed fetuses and heart weight, between groups receiving quercetin alone and quercetin plus theophylline and the control group. Moreover, quercetin negated the teratogenic effect of theophylline (increased percentage of absorbed fetuses and fetal heart weight).

 In Lindstrom et al. study, following the theophylline administration with the doses of124, 218 and 259 mg/kg, during the 6th to 15th days of rats’ pregnancy, maternal womb weight decrease was observed in a dose dependent manner. Moreover, the number of fetuses decreased by 0.4% and their weight decreased by 0.3 and 0.4% in males and females, respectively. However, no macroscopic anomaly was observed. The result of our study is consistent with the above study (4).

Matsuoka et al. (6) indicated that the oral administration of caffeine (a metabolite of theophylline) with the dose of 150 mg/kg on the 11th day of rats’ pregnancy, 87.7 % of fetuses suffered from cardiac anomalies, the most important of which was VSD. Moreover, skeletal malformations were observed in these fetuses. This result is inconsistent with that of the present research.

Cardiac anomalies including DORV, heart murmur, aortic displacement, VSD, pulmonary valve stenosis and hypoplasia of the left ventricle were observed in a female infant, born to a 19-year-old mother who had consumed 900 mg/kg of oral theophylline, daily, since the age of 7 due to asthma. Moreover, cardiac failure, hypoplasia of the ascending aorta, aortic and left ventricle arch, aortic valve atresia and heart hypoplasia syndrome were observed in an infant born to a 32-year-old mother with asthma, who had used theophylline, orally, 300 mg/kg, twice a day (2).

Cardiac conditions, observed in the above studies are inconsistent with the results of the present research, which can be due to using animal models. The hypothesis that the consumption of antioxidants can reduce fetal complications of drugs is confirmed in other studies. This result is rather consistent with that of the present study.

Administration of vitamins A, C and E as antioxidants in the 9th and 10th days of rats’ pregnancy can prevent nitrophen-induced conditions like the heart tissue hypoplasia, accompanied with diaphragmatic hernia. In this study, the administration of vitamins A, C and E prevented decreased fetal weight and cell count and increased apoptosis (12).

The administration of vitamin E was observed to have preventive effects onnitrophen-induced pulmonary hypoplasia in rat fetuses (13). In this study, the serum malondialdehyde level was significantly higher in the group receiving theophylline, compared to other groups. Since malondialdehyde is the final composition of lipid peroxidation and indicates the sensitivity of lipids to oxidation, its increase is indicative of increased oxidation level in theophylline receiving rats. Decreased malondialdehyde level in the theophylline and quercetin receiving group is indicative of quercetin ability, in increasing the antioxidant and decreasing the lipid oxidation levels.

Another study indicated that the administration of quercetin (15 mg/kg) for four weeks, decreases the serum malondialdehyde level in streptozotocin-induced diabetic rats (14). Another study indicated that the administration of quercetin in rats with monosodium urate-induced gout, decreases serum malondialdehyde level and increases serum antioxidant enzymes (15). Annapurna et al. (16) stated that the administration of quercetin (50 mg/kg), ten minutes before stroke, decreases the malondialdehyde level in rats. Atef et al. (17) observed that daily oral administration of 20 mg/kg of quercetin for seven consecutive days, significantly decreases the malondialdehyde level in diabetic rats (18).

In this study, the comparison of average glutathione peroxidase level indicated that the amount of this enzyme, in the theophylline receiving group is lower than the other groups. It is also significantly higher in the group receiving quercetine. Decreased glutathione peroxidase level in the theophylline receiving group is induced by increased oxidation process. The glutathione peroxidase level in the quercetin receiving group and lack of a significant difference, regarding this level, between the group receiving quercetin and the one receiving both theophylline and quercetin is related to quercetin antioxidant properties. The result of the present study is consistent with that of the other studies.

Quercetin increases the glutathione peroxidase activity gene expression in the liver of old rats (19). Moreover, the administration of quercetin to little white mice with the daily doses of 75, 50 and 25 mg/kg, for 15 consecutive days, increases the glutathione peroxidase level (20).

A study indicated that intraperitoneal administration of 50 mg/kg of quercetin 10 minutes before ischemia, increases the glutathione peroxidase level, in rats (21, 22). Moreover, oral administration of 10 mg/kg of quercetin to rats with hypertension increases the glutathione peroxidase activity (23).

The antioxidant effect of quercetin on rats was investigated in another study, using hepatocyte cultivation. To induce oxidative stress, H_2_O_2_ was used. They concluded that the quercetin antioxidant activity is due to the glutathione peroxidase activation (24). Quercetin is a natural antioxidant, preventing free radicals formation and applies its antioxidant effect by inhibiting lipid peroxidation, blocking xanthine oxidase, chelating iron and eliminating free radicals.

The role of quercetin in decreasing the teratogenic effects of theophylline in rat embryos was demonstrated in this study for the first time. Regarding the role of quercetin in the elimination of free radicals and oxidative stress, it seems that as an antioxidant, quercetin can decrease some of the teratogenic effects of theophylline and also has an effect in the prevention of fetal complications. Further studies on different animal models and ultimately adapting the existing laws and regulations to human models are essential to ensure the beneficial effects of quercetin.

## References

[1] Watanabe S, Yamakami J, Tsuchiya M, Terajima T, Kizu J, Hori S (2008). Anti-inflammatory effect of theophylline in rats and its involvement of the glucocorticoid-glucocorticoid receptor system.. J Pharmacol Sci..

[2] Park JM, Schmer V, Myers TL (1990). Cardiovascular anomalies associated with prenatal exposure to theophylline.. South Med J..

[3] Goodman LS, Brunton L, Chabner B, Knollmann BC (2011). Goodman and Gilman's The Pharmacological Basis of Therapeutics..

[4] Lindstrom P, Morrissey RE, George JD, Price CJ, Marr MC, Kimmel CA (1990). The developmental toxicity of orally administered theophylline in rats and mice.. Fundam Appl Toxicol..

[5] Labovitz E, Spector S (1982). Placental theophylline transfer in pregnant asthmatics.. JAMA..

[6] Matsuoka R, Gilbert EF, Bruyere HJ Jr, Fang TT, Nishikawa T, Opitz JM (1987). Experimentally induced cardiovascular malformations in the chick embryo. Part II. Teratogenic effect of Tedral (theophylline, ephedrine, and phenobarbital) on cardiac development in chick embryos.. Birth Defects Orig Artic Ser..

[7] Shibata M, Wachi M, Kawaguchi M, Kojima J, Onodera K (2000). Teratogenic and fetal toxicity following intravenous theophylline administration in pregnant rabbits is related to maternal drug plasma levels.. Methods Find Exp Clin Pharmacol..

[8] Overman DO, Beaudoin AR (1971). Early biochemical changes in the embryonic rat heart after teratogen treatment.. Teratology..

[9] Defoort EN, Kim PM, Winn LM (2006). Valproic acid increases conservative homologous recombination frequency and reactive oxygen species formation: a potential mechanism for valproic acid-induced neural tube defects.. Mol Pharmacol..

[10] Khaki A, Fathi AF, Ahmadi Ashtiani HR, Rezazadeh S, Rastegar H, Imani AM (2010). Compartments of quercetin & allium cepa (onion) on blood glucose in diabetic rats.. J Med Plants..

[11] Asla BH, Delazarb A, Assadyb M (2006). Effects of Quercetin and ACTH on Morphine-Induced Tolerance and Dependence in Mice.. Iran J Pharm Sci..

[12] Gonzalez-Reyes S, Martinez L, Tovar JA (2005). Effects of prenatal vitamins A, E, and C on the hypoplastic hearts of fetal rats with diaphragmatic hernia.. J Pediatr Surg..

[13] Islam S, Narra V, Cote GM, Manganaro TF, Donahoe PK, Schnitzer JJ (1999). Prenatal vitamin E treatment improves lung growth in fetal rats with congenital diaphragmatic hernia.. J Pediatr Surg..

[14] Huang J, Zhu M, Tao Y, Wang S, Chen J, Sun W (2012). Therapeutic properties of quercetin on monosodium urate crystal-induced inflammation in rat.. J Pharm Pharmacol..

[15] Annapurna A, Ansari MA, Manjunath PM (2013). Partial role of multiple pathways in infarct size limiting effect of quercetin and rutin against cerebral ischemia-reperfusion injury in rats.. Eur Rev Med Pharmacol Sci..

[16] Annapurna A, Ansari MA, Manjunath PM (2013). Partial Role of Multiple Pathwys in Infarct Size Limitation Effect of Querecentin and Rutin Against Cerebral Ischemia-Repeefusion in Rats.. Eur Rev Med Pharmacol Sci..

[17] Atef E, El-Baky A (2011). Quercetin protective action on oxidative stress, Sorbitol insulin resistance and cells function in experimental diabetic rats.. Int J Pharm Sci Res..

[18] Habib HZ, Louka ML, Nassef NAH (2013). In vivo effect of quercetin flavonoid on hepatic gene expression and enzyme activity levels in old rats.. Egypt J Biochem Mol Biol..

[19] Molina MF, Sanchez-Reus I, Iglesias I, Benedi J (2003). Quercetin, a flavonoid antioxidant, prevents and protects against ethanol-induced oxidative stress in mouse liver.. Biol Pharm Bull..

[20] Kahraman A, Inal ME (2002). Protective effects of quercetin on ultraviolet A light-induced oxidative stress in the blood of rat.. J Appl Toxicol..

[21] Inal M, Altinisik M, Bilgin MD (2002). The effect of quercetin on renal ischemia and reperfusion injury in the rat.. Cell Biochem Funct..

[22] Duarte J, Galisteo M, Ocete MA, Perez-Vizcaino F, Zarzuelo A, Tamargo J (2001). Effects of chronic quercetin treatment on hepatic oxidative status of spontaneously hypertensive rats.. Mol Cell Biochem..

[23] Nagata H, Takekoshi S, Takagi T, Honma T, Watanabe K (1999). Antioxidative action of flavonoids, quercetin and catechin, mediated by the activation of glutathione peroxidase.. Tokai J Exp Clin Med..

[24] Harwood M, Danielewska-Nikiel B, Borzelleca JF, Flamm GW, Williams GM, Lines TC (2007). A critical review of the data related to the safety of quercetin and lack of evidence of in vivo toxicity, including lack of genotoxic/carcinogenic properties.. Food Chem Toxicol..

